# A rare case of myxoma presenting as a scrotal lump

**DOI:** 10.1016/j.radcr.2023.10.073

**Published:** 2023-11-24

**Authors:** Muad Abdi Hassan, Jouhar J. Kolleri, Noor Al-Nassr, Tala Kanaan, Khaled A. Murshed, Nabil Sherif Mahmood

**Affiliations:** a Clinical Imaging Department, Hamad Medical Corporation, Doha, Qatar; b Histopathology Department, Hamad Medical Corporation, Doha, Qatar

**Keywords:** Scrotum, Myxoma, Scrotal wall myxoma, Cold cyst

## Abstract

A myxoma is a benign intramuscular gelatinous tumor that is rarely known to arise from the scrotum. It can be confused both clinically and radiologically with other more common scrotal wall lesions such as a sebaceous cyst or an abscess. We report a case of a 54-year-old patient who presented with a scrotal wall swelling that was initially suspected to be an infected cyst or cold abscess on imaging. The final diagnosis of a myxoma was made after surgical excision and histopathological examination.

## Introduction

A benign intramuscular gelatinous tumor called a myxoma commonly manifests as an intramuscular lump. It is a class of mesenchymal tumors mostly composed of extracellular myxoid matrix. Most myxoma patients are in their fifth or sixth decade of life [1,2]. Myxomas can be classified as intramuscular myxoma, superficial angiomyxoma, aggressive angiomyxoma, myelolipoma, acral fibro myxoma, and dermal myxoma based on radiological and histological characteristics. Intramuscular myxoma is the most common type [3]. It is an uncommon tumor with a frequency ranging between 0.10 and 0.13 per 100,000 persons [4]. The swelling is often isolated, nontender, firm, and fluctuant steadily increasing in size. Although it may affect any muscle in the body, it often affects the thighs, buttocks, and shoulders [5]. Presentation as an incidental scrotal wall lump is exceedingly rare. A variety of imaging techniques, including magnetic resonance imaging (MRI), computed tomography (CT), and ultrasound may be used to image myxomas [5]. Histopathological confirmation is often required as the imaging findings may not be conclusive [6]. Surgical excision with clean margins is the preferred treatment of choice. Local recurrence may result from insufficient excision. Recurrence is however quite uncommon [6].

## Case presentation

A 54-year-old man with a medical history of hypertension, coronary artery disease requiring previous percutaneous coronary intervention, dyslipidemia, and a history of smoking presented to the clinic with a scrotal wall lesion noticed for a few months, progressively increasing in size. He denied any testicular pain or urinary symptoms. On physical examination, a firm 3 cm lesion arising from the left scrotal wall with a narrow neck was palpated. The testes and epididymis appeared normal. Due to the concerning nature of the lesion, an urgent scrotal ultrasound was ordered for further evaluation.

**Fig. 1:**
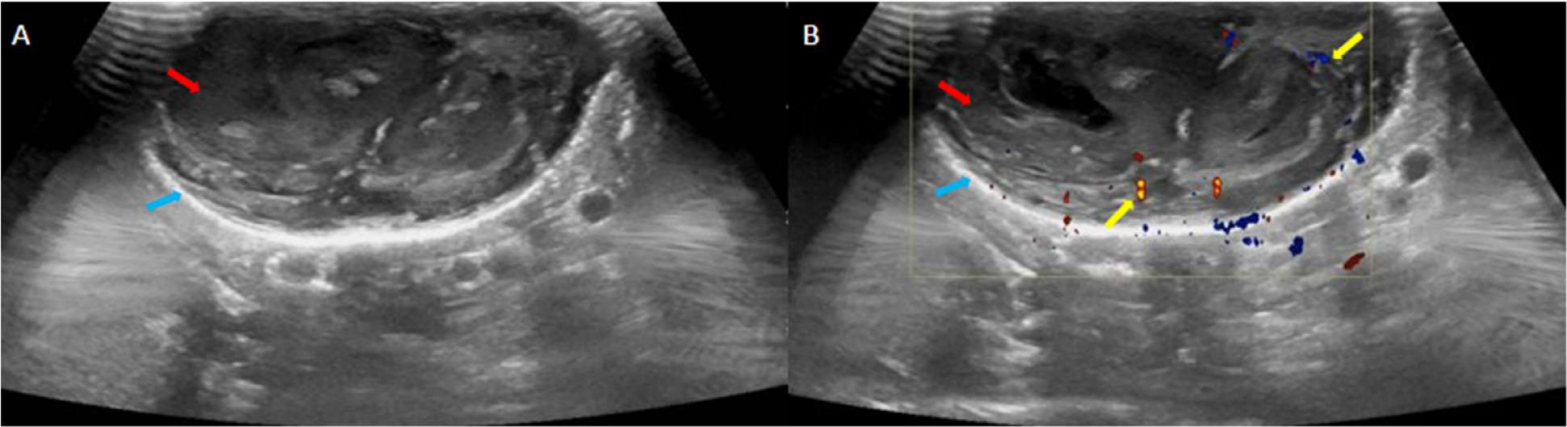
Ultrasound scrotum (A & B) showing a well-defined lesion of mixed echotexture in the scrotal wall (Red arrows), with an echogenic wall (blue arrows) and necrotic or turbid internal components. The solid-appearing components show areas of increased vascularity on the color Doppler (yellow arrows).

Ultrasound of the scrotum was done, which demonstrated a well-circumscribed abnormality of mixed echotexture at the site of concern in the scrotal wall. It measured around 4 × 2 cm in size. The lesion had an echogenic wall and internal necrotic or turbid components with vascularity noted within the solid-appearing elements. Both testes and epididymis showed normal size, echotexture, and vascularity. No hydrocele was seen (Fig. 1). An inflammatory pathology such as a cold abscess or an infected cyst was considered among the differentials, however, the possibility of a soft tissue neoplasm could not be ruled out either. Histopathological correlation was hence suggested.

Given the concerning findings on ultrasound, the patient underwent surgical excision of the scrotal wall lesion under local anesthesia. The excised specimen was sent for histopathological analysis.

**Fig. 2:**
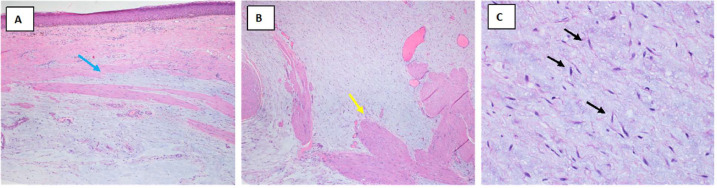
Histopathologic features. (A) Photomicrograph showing the scrotal epidermis with an underlying myxoid lesion (blue arrow) located in the dermis (hematoxylin and eosin stain x40). (B) the myxoid lesion is hypocellular and hypovascular, and dissecting into the muscle fibers (yellow arrow) (hematoxylin and eosin stain x100). (C) High power view shows bland-looking stellate to spindle cells (black arrows) embedded in the myxoid stroma (hematoxylin and eosin stain x400).

Histopathology showed a scrotal wall lesion dissecting in between the muscle fibers composed of bland spindle to stellate cells embedded in myxoid stroma. Few associated small blood vessels were noted. Immunohistochemical staining showed that the spindle cells were immunoreactive for smooth muscle actin and calponin, with focal expression of estrogen receptor (ER) and desmin. They were negative for CD34, S100, Cytokeratin AE1/AE3, epithelial membrane antigen (EMA), STAT6, MDM2, and CDK4. The Ki-67 proliferation index was approximately 5% (Fig. 2). Based on the morphologic and immunophenotypic characteristics, the diagnosis of scrotal wall myxoma was rendered.

The patient's clinical progress and response to treatment have been satisfactory, with no evidence of recurrence or complications noted during the follow-up period.

## Discussion

This is a rare case of a myxoma presenting as a scrotal wall lump thus clinically mimicking other scrotal lumps such as a scrotal wall abscess, sebaceous cyst, or sometimes hydrocele or varicocele. Although myxomas may affect any muscle in the body, they most frequently affect the thighs, buttocks, and shoulders. Involvement of the scrotum is exceedingly rare.

From a clinical perspective, an intramuscular myxoma often presents as a solitary, discrete, painless, and hard palpable swelling [1].

On ultrasound, intramuscular myxomas are usually hypo-echoic with well-defined margins, sometimes containing anechoic cystic foci within. The presence of anechoic cystic internal contents may make them mimic necrotizing infectious or inflammatory processes such as abscesses or infected cysts. CT usually shows a well-defined homogeneous low-density enhancing lesion. On MRI, the lesion usually appears as a well-defined and smooth intramuscular lump that is homogeneously hypointense on T1-weighted images, and extremely hyperintense on T2-weighted images, similar to fluid signal. The presence of a perilesional rind of fat or edema has been suggested as a definite sign to diagnose myxoma on MRI [3].

Histologically, myxoma is typically a hypovascular and hypocellular myxoid lesion composed of the bland spindle to stellate-shaped cells. No necrosis or mitotic activity should be present. If the lesion is more cellular and hyper-vascular, another differential diagnosis should be considered like myxofibrosarcoma, myxoid liposarcoma, and low-grade fibro myxoid sarcoma. Careful histological examination and tissue sampling are essential to reach a definitive diagnosis [7].

They are treated with surgical resection ensuring clear margins. The likelihood of recurrence is relatively low [8].

## Conclusion

A myxoma presenting as a scrotal wall lump is exceedingly rare and can mimic more common pathologies such as a cold abscess or an infected sebaceous cyst. A high index of suspicion is required especially when the imaging shows a mixed echogenic mass with internal cystic changes.

## Ethics approval and consent to participate

The article describes a case report. Therefore, no additional permission from our Ethics Committee was required.

## Availability of data and material

All data generated or analyzed during this study are included in this published article.

## Authors' contributions

Data Collection, Literature Search, Manuscript Preparation (draft and final editing): MAH, JJK, NAN, TK, KAM, NSM

All authors read and approved the final manuscript.

## Patient consent

We obtained written informed consent from the patient to publish this case report by the journal's consent policy.
